# Upper limb gangrene due to a subclavian arterial thrombosis associated with thoracic outlet syndrome following cervical spondylotic amyotrophy

**DOI:** 10.1093/jscr/rjab495

**Published:** 2021-11-11

**Authors:** Tsuyoshi Yamada, Toshifumi Kudo, Atsuyuki Kawabata, Toshitaka Yoshii

**Affiliations:** Department of Orthopaedic Surgery, Kudanzaka Hospital, Tokyo, Japan; Department of Orthopaedic Surgery, Tokyo Medical and Dental University, Tokyo, Japan; Department of Specialized Surgery, Tokyo Medical and Dental University, Tokyo, Japan; Department of Orthopaedic Surgery, Tokyo Medical and Dental University, Tokyo, Japan; Department of Orthopaedic Surgery, Tokyo Medical and Dental University, Tokyo, Japan

## Abstract

Thoracic outlet syndrome (TOS) refers to an abnormal compression of the subclavian vessels and the brachial plexus at the base of the neck or thoracic outlet. The authors described a novel case of a 66-year-old male with arterial TOS and unilateral upper rib dysplasia following cervical spine disease. Owing to the comorbidity of cervical spondylotic amyotrophy and a history of sloping shoulder due to rib dysplasia, TOS occurred, which subsequently led to subclavian arterial thrombosis and eventually resulted in the gangrene of the hand. Thrombectomy and surgical resection of the first and second ribs avoided the progression of further neurovascular disorder.

When treating a cervical spondylotic patient, concurrent TOS can be obscured by symptoms caused by cervical lesions, leading to difficulty in identifying the origin of these neurovascular findings. The possibility of subsequent thromboembolic events based on TOS should be considered, especially in cases with dysplasia of the upper ribs.

## INTRODUCTION

Thoracic outlet syndrome (TOS) refers to an abnormal compression of the subclavian vessels and the brachial plexus at the base of the neck or thoracic outlet. There are various symptoms and multiple potential etiologies of TOS, such as cervical rib, abnormal ligamentous tissue, the scalenus pleuralis and aneurysms of the subclavian artery [1, 2]. Sometimes cervical ribs produce successive distal arterial embolism, resulting in severe ischemia of the affected upper extremity [3]. In the first place, a thromboembolism in an upper limb itself is a rare event; however, when diagnosed promptly, it can be successfully treated with surgery [4]. The signs of ischemic changes in the upper limbs can be masked by other circumstances, such as comorbidity, perioperative conditions or complications [5].

The authors report a rare case of arterial TOS with unilateral rib dysplasia following cervical spine disease.

## CASE REPORT

**Figure 1:**
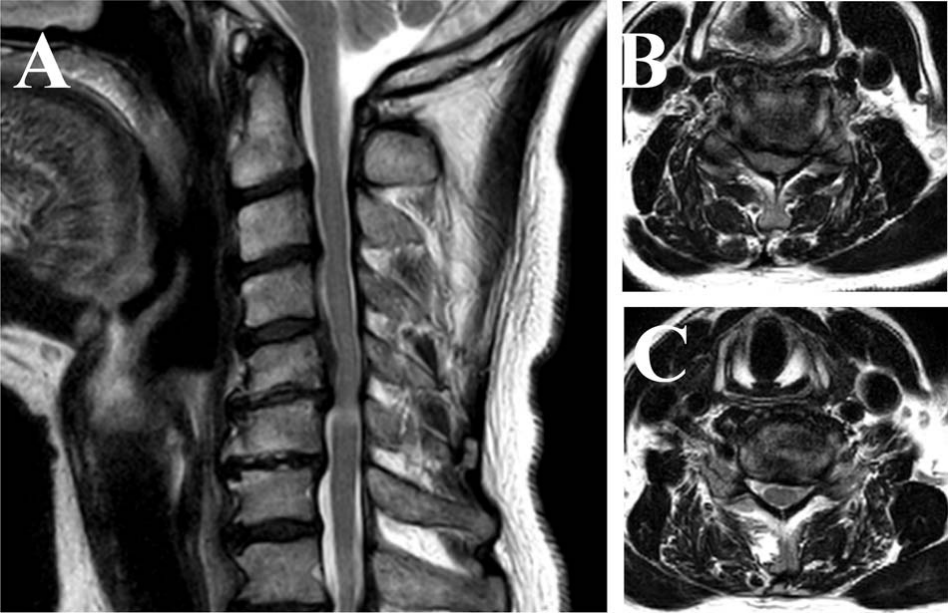
Magnetic resonance imaging of the cervical spine (T2-weighted image). (**A**) Sagittal view images showed that compressive lesions in the cervical spine were located at the C5-C6 levels. The high signal lesion in the spinal cord was seen at the C5-C6 level. (**B**) Axial view of the C5-C6 level showed the spinal cord compression in the canal. (**C**) Axial view of the C6-C7 level showed the stenosis of the left side of foramen.

A 66-year-old Japanese male complained of weakness in his left fingers and hand, with neither gait impairment nor any symptoms in the lower extremities 1 year ago. Compressive lesions in the cervical spine were located at the C5-C7 level (Fig. 1), leading to the diagnosis as distal-type cervical spondylotic amyotrophy (CSA). Although the manual muscle test (MMT) of his left hand was grade 2, he preferred conservative therapy as his right hand was dominant.

**Figure 2:**
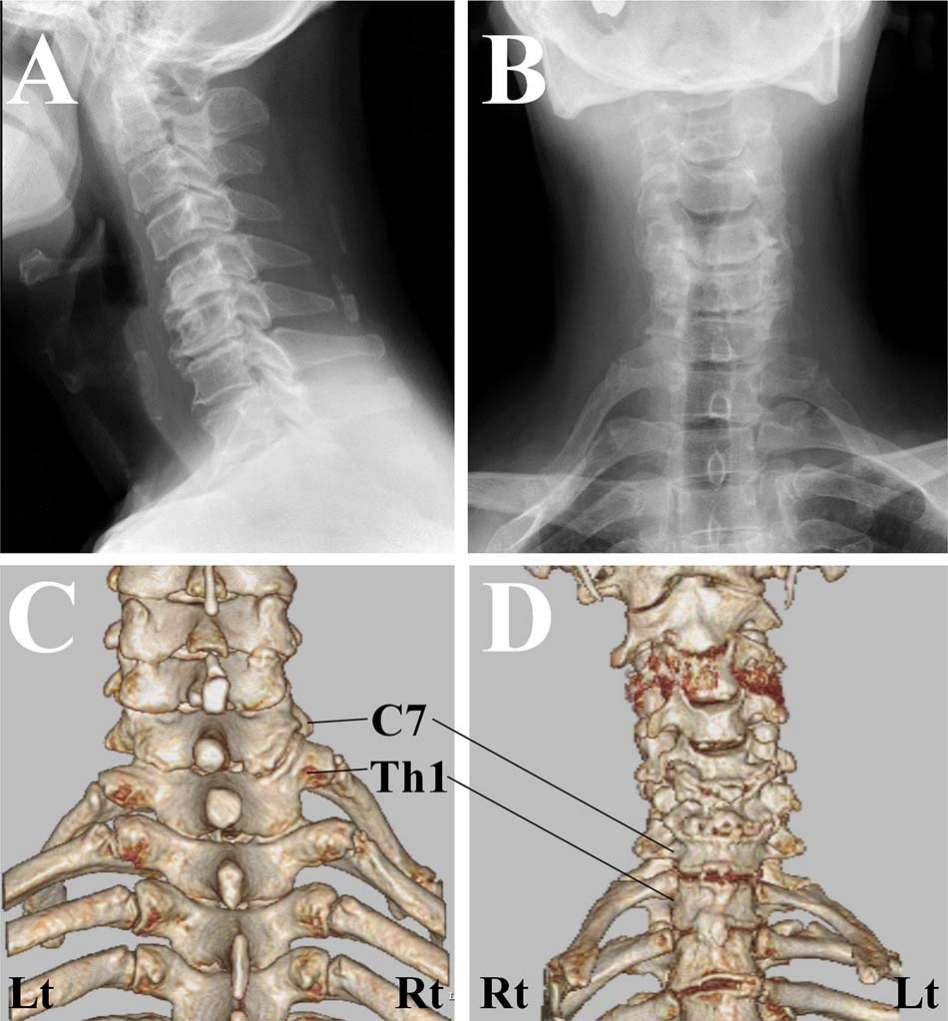
Cervical spine X-ray and computed tomography (CT) images. (**A**) X-rays image of lateral view showed the straight alignment of the cervical spine. (**B**) X-rays image of anterior–posterior view showed bilateral sloping shoulders. (**C**) CT image of posterior–anterior view. The first rib on the right side originated from the higher position of the Th1 vertebra than that on the left side. (**D**) CT image of anterior–posterior view. CT revealed the existence of the joint between the first rib and the second rib on the right side.

**Figure 3:**
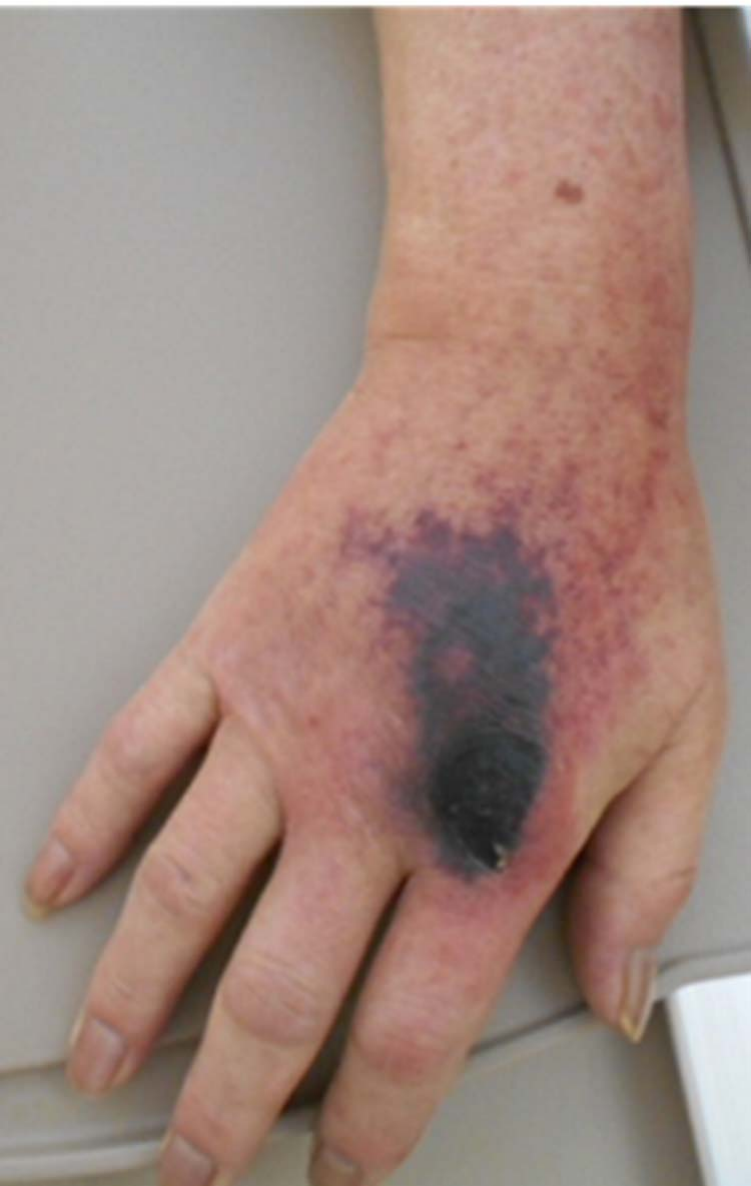
Macro-image of the right hand. The gangrene and inflammation developed 3 weeks after the onset of the paralysis in the right hand.

One year after the onset of symptoms in his left hand, he complained of weakness in his right fingers and hand (MMT grade 2) without any pain/numbness. We confirmed the pulses of the radial artery at this original presentation. Magnetic resonance imaging showed a more severe compressive lesion at the C5-C6 level from 1 year ago, while computed tomography (CT) revealed that there was a difference between the attachment to the Th1 vertebrae between the right and left first ribs, and the existence of the joint between the first rib and the second rib (Fig. 2). Given his past history and his clinical/radiological findings, the symptoms were compatible with CSA. However, 2 weeks after the right-hand paresis, his sloping shoulder, as well as weak pulse of the radial artery, pain and gangrene in his right hand gradually developed probably due to concurrent TOS (Fig. 3).

**Figure 4:**
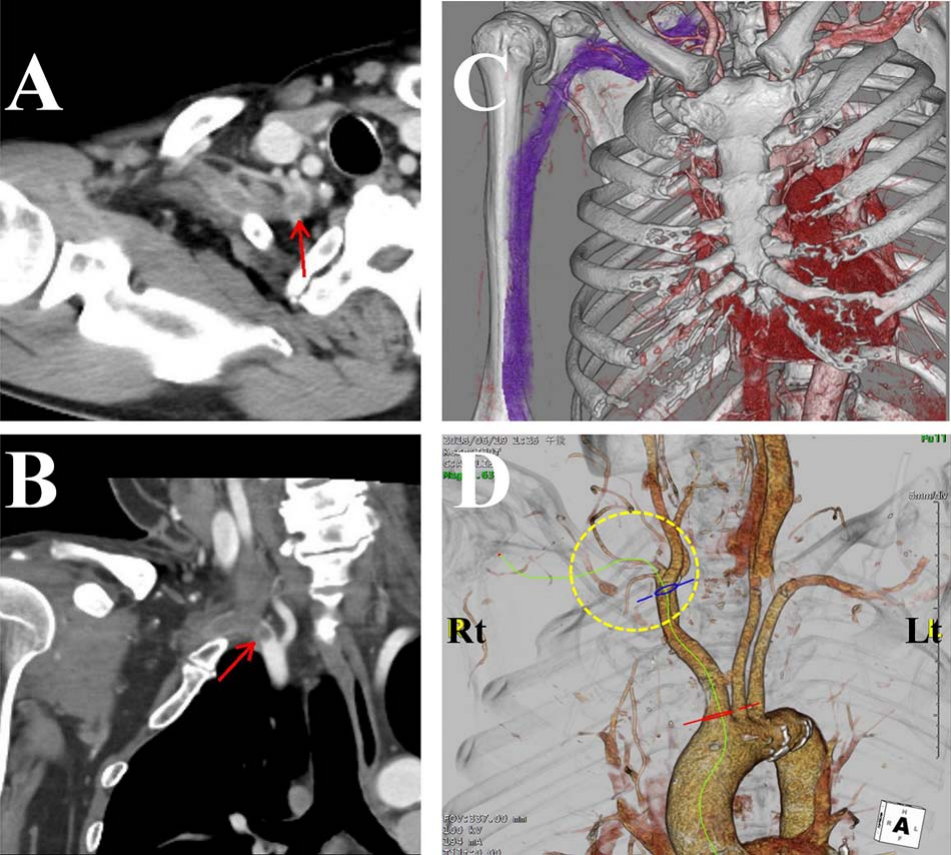
Contrast-enhanced computed tomography (CT) images of the thorax. (**A**) Axial view. (**B**) Coronal view. (**C**, **D**) Three-dimensional CT images. The subclavian artery (circle) was occluded due to a thrombus (arrow) at the level of the dysplasia of ribs.

The laboratory data revealed inflammation (white blood cell: 17 500/μl; C-reactive protein: 19.34 mg/dl). Although an increase in D-dimer levels (1.1 ng/ml) was not observed, the contrast-enhanced CT imaging revealed that the subclavian artery was occluded, and a successive distal arterial embolism was produced (Fig. 4). We diagnosed him with an acute arterial occlusion associated with TOS.

**Figure 5:**
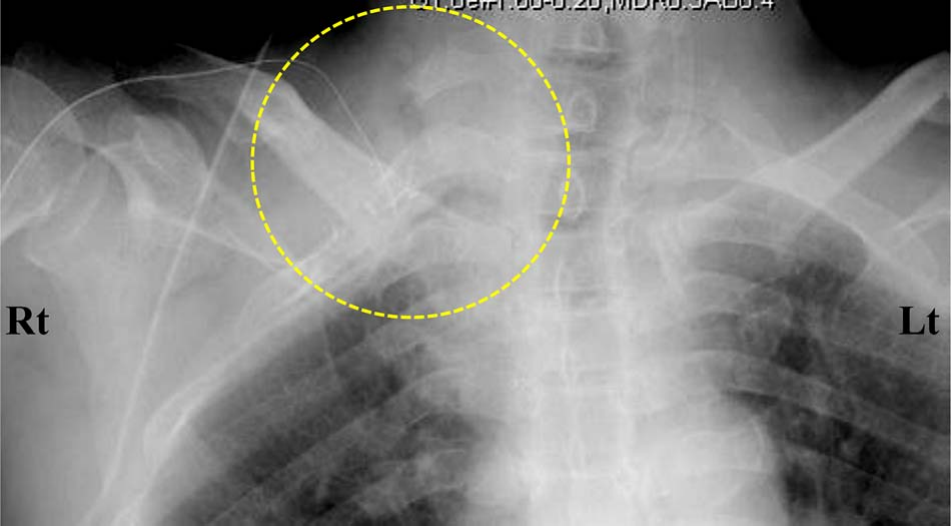
Postoperative cervicothoracic spine X-ray. Anterior–posterior view. The right first and second ribs were surgically resected (circle).

Three weeks after the onset of right-hand symptoms, he was treated with an emergency thrombectomy. The right subclavian arterial lumen was filled with an organized thrombus. After a few days of anticoagulation therapy (heparin 20 000 U/day) to prevent recurrent or new emboli, the pulse of the right radial artery could be felt, and the gangrene seemed to be reduced. One month after thrombectomy, surgical decompression was performed. After the scalenus anterior/medius muscle was dissected free from the first/second rib using the supraclavicular fossa approach, the high riding first rib was resected with the second rib (Fig. 5).

Although the paresis in his right hand partially remained, we were able to avoid amputation. The patient showed tolerable upper extremity function without sensory deficit in the right hand 1 year after thrombectomy, and he could go to work at his same place of employment.

## DISCUSSION

While the most frequent type of TOS is neurogenic, and arterial and venous TOSs are relatively uncommon, they can coexist and possibly overlap [6]. Clinicians frequently have difficulty in categorizing them clearly, especially in cases with cervical spondylotic lesions. The cases with arterial TOS that required revascularization due to gangrene of the fingers and/or hand have been reported in only a few articles [3, 7]. These severe cases of TOS, which could be caused by cervical rib abnormalities, and could sometimes result in paralysis or amputation of the hand, emphasize the importance of early diagnosis of this condition.

In most upper limb thromboembolism cases, however, it is not difficult to detect the thromboemboli by using duplex imaging, Doppler ultrasonography and angiography [8]. In our case, the obstruction was at the subclavian artery level, consistent with previous severe TOS cases with gangrene [3, 7]. The paresis and ischemic changes in the hands owing to TOS/CSA seemed to coincide with each other. To begin with, although the cervical rib is one of the most common causes of TOS, the high riding first rib or the joint between the first rib and the second rib is an exceptionally rare cause. Secondly, CSA itself is a rare form of cervical spondylotic myelopathy, in which the clinical status before surgery (symptom duration and preoperative MMT grade) significantly influences the surgical outcome [9]. In the present case, although the conservative therapy was continued for 1 year, weakness in the contralateral hand still developed. The uncommon comorbidity of CSA and a sloping shoulder originating from the dysplasia of ribs could be the trigger for severe TOS, followed by subclavian arterial thrombosis and gangrene of the right hand. The signs of ischemic changes in the upper limbs were partially masked by the paresis due to CSA. Lack of pulse in the radial artery of the right hand could be attributed to the arterial thrombosis.

The patient eventually developed a fever, and further gangrene in his right hand. The limb can remain compromised once the ischemic cascade has been initiated, even after successful revascularization. Despite the late presentation due to these extremely rare comorbidities, thrombectomy and surgical treatment avoided the progression of further neurovascular disorder.

TOS and cervical stenosis can coexist and possibly overlap. When treating a cervical spondylotic patient with palsies in the upper limbs, although rare, we should consider the possible existence of concurrent TOS behind the cervical lesions. The possibility of subsequent thromboembolic events based on TOS also should be considered, especially in the cases with abnormal ribs. Rapid diagnosis and surgical intervention for the circulatory disorder are essential for preventing the catastrophic events associated with neurovascular disorder.

## References

[1] Gruss JD , Bartels D, Vargas H, Ohta T, Tsafandakis E, Schlechtweg B, et al. Shoulder girdle compression syndrome. J Cardiovasc Surg (Torino) 1982;23:221–4. 7085741

[2] Davidović LB , Lotina SI, Jakovljević NS, Pavlović GS, Lj K. Aneurysms of the subclavian artery. Srp Arh Celok Lek 2000;128:184–90. 11089419

[3] Shindo S , Kamiya K, Suzuki O, Kobayashi M, Tada Y. Distal arterial reconstruction using Esmarch's bandage technique to salvage upper extremity function in thoracic outlet syndrome caused by cervical ribs: a report of two cases. Surg Today 1994;24:1107–10. 7780238 10.1007/BF01367467

[4] Wirsing P , Andriopoulos A, Botticher R. Arterial embolectomies in the upper extremity after acute occlusion. Report on 79 cases. J Cardiovasc Surg (Torino) 1983;24:40–2. 6833351

[5] Yamada T , Yoshii T, Yoshimura H, Suzuki K, Okawa A. Upper limb amputation due to a brachial arterial embolism associated with a superior mesenteric arterial embolism: a case report. BMC Res Notes 2012;5:372. 22828325 10.1186/1756-0500-5-372 PMC3410779

[6] Stilo F , Montelione N, Benedetto F, Spinelli D, Vigliotti RC, Spinelli F. Thirty-year experience of transaxillary resection of first rib for thoracic outlet syndrome. Int Angiol 2020;39:82–8. 31814380 10.23736/S0392-9590.19.04300-1

[7] Bucek RA , Schnürer G, Ahmadi A, Maca TH, Meissl G, Minar E. A severe case of vascular thoracic outlet syndrome. Wien Klin Wochenschr 2000;112:973–7. 11142135

[8] Panetta T , Thompson JE, Talkington CM, Garrett WV, Smith BL. Arterial embolectomy: a 34-year experience with 400 cases. Surg Clin North Am 1986;66:339–53. 3952607 10.1016/s0039-6109(16)43886-7

[9] Inui Y , Miyamoto H, Sumi M, Uno K. Clinical outcomes and predictive factors relating to prognosis of conservative and surgical treatments for cervical spondylotic amyotrophy. Spine (Phila Pa 1976) 2011;36:794–9. 20736889 10.1097/BRS.0b013e3181e531a1

